# From basic research to clinical development of MEK1/2 inhibitors for cancer therapy

**DOI:** 10.1186/1756-8722-3-8

**Published:** 2010-02-11

**Authors:** Christophe Frémin, Sylvain Meloche

**Affiliations:** 1 Institut de Recherche en Immunologie et Cancérologie and Departments of Pharmacology and Molecular Biology, Université de Montréal, Montreal, Quebec H3C 3J7, Canada

## Abstract

The Ras-dependent Raf/MEK/ERK1/2 mitogen-activated protein (MAP) kinase signaling pathway is a major regulator of cell proliferation and survival. Not surprisingly, hyperactivation of this pathway is frequently observed in human malignancies as a result of aberrant activation of receptor tyrosine kinases or gain-of-function mutations in *RAS *or *RAF *genes. Components of the ERK1/2 pathway are therefore viewed as attractive candidates for the development of targeted therapies of cancer. In this article, we briefly review the basic research that has laid the groundwork for the clinical development of small molecules inhibitors of the ERK1/2 pathway. We then present the current state of clinical evaluation of MEK1/2 inhibitors in cancer and discuss challenges ahead.

## Introduction

Human tumorigenesis is a multistep process during which accumulation of genetic and epigenetic alterations leads to the progressive transformation of a normal cell into a malignant cancer cell. During this process, cancer cells acquire new capabilities (hallmarks) that enable them to escape from normal homeostatic regulatory defense mechanisms. These hallmarks are defined as: self-sufficiency in growth signals, insensitivity to antiproliferative signals, evasion from apoptosis, limitless replicative potential, sustained angiogenesis, and increased motility and invasiveness [1]. While the mechanisms by which cancer cells acquire these capabilities vary considerably between tumors of different types, most if not all of these physiological changes involve alteration of signal transduction pathways. Among the signaling pathways most frequently dysregulated in human cancer is the Ras-Raf-MEK-extracellular signal-regulated kinase 1 and 2 (ERK1/2) pathway.

**Figure 1:**
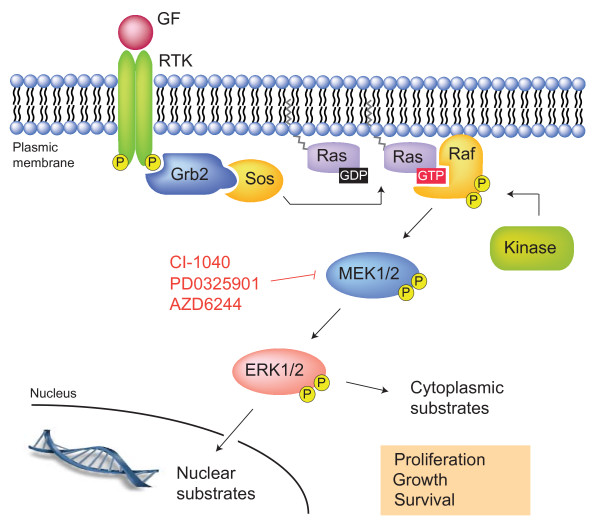
**Schematic representation of the Ras-Raf-MEK-ERK1/2 MAP kinase pathway**. The figure shows the cascade of activation of the MAP kinases ERK1/ERK2 mediated by growth factor binding to receptor tyrosine kinases. See text for details. GF, growth factor; RTK, receptor tyrosine kinase.

The Ras-dependent ERK1/2 mitogen-activated protein (MAP) kinase pathway is one of the best-studied signal transduction pathways (Fig. 1). Since the discovery of MAP kinases by Ray and Sturgill in 1988 [2], more than 11,000 articles have been published on this topic. ERK1/2 MAP kinases are activated by virtually all growth factors and cytokines acting through receptor tyrosine kinases, cytokine receptors or G protein-coupled receptors. Typically, ligand binding to receptor tyrosine kinases induces dimerization of the receptor and auto-phosphorylation of specific tyrosine residues in the C-terminal region. This generates binding sites for adaptor proteins, such as growth factor receptor-bound protein 2 (GRB2), which recruit the guanine nucleotide exchange factor Sos at the plasma membrane. Sos activates the membrane-bound Ras by catalyzing the replacement of GDP with GTP. In its GTP-bound form, Ras recruits Raf kinases (ARAF, BRAF and CRAF) to the plasma membrane, where they become activated by a complex interplay of phosphorylation events and protein-protein interactions. Raf acts as a MAP kinase kinase kinase (MAPKKK) and activates the MAP kinase kinases (MAPKKs) MEK1 and MEK2, which, in turn, catalyze the activation of the effector MAP kinases ERK1 and ERK2 [3]. Once activated, ERK1/ERK2 phosphorylate a panoply of nuclear and cytoplasmic substrates involved in diverse cellular responses, such as cell proliferation, survival, differentiation, motility, and angiogenesis [4].

## MEK1/MEK2 and the family of MAP kinase kinases

**Figure 2:**
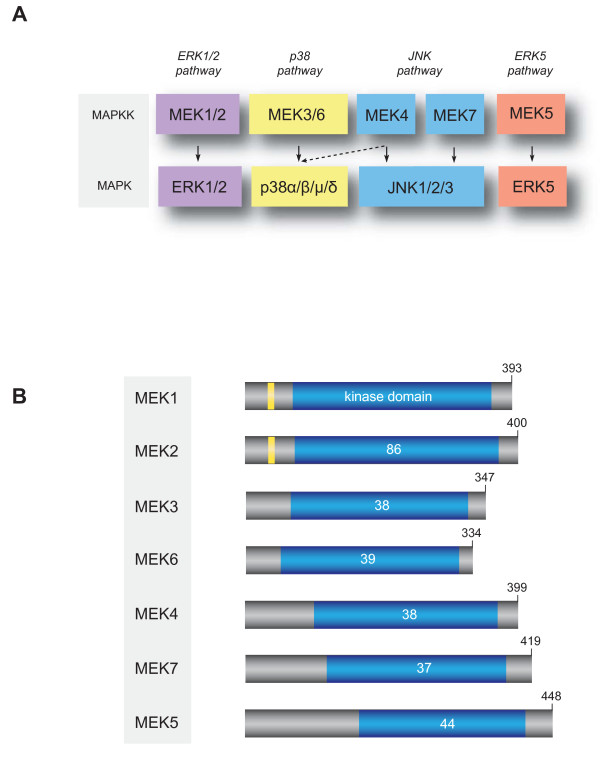
**The MAP kinase kinases family**. (A) MAP kinases and their upstream MAPKKs. (B) Schematic representation of human MAPKKs. MAPKKs are composed of a kinase catalytic domain (in blue) flanked by N- and C-terminus extensions of varying lengths. The percentage of identity of the kinase domain with MEK1 is indicated. An NES, only present in MEK1 and MEK2, is indicated in yellow.

MEK1 and MEK2 belong to the family of MAPKKs (also known as MEKs or MKKs), which are dual specificity enzymes that phosphorylate threonine and tyrosine residues within the activation loop of their MAP kinase substrates [5]. The human genome encodes seven MAPKK enzymes that regulate the activity of four distinct MAP kinase pathways (Fig. 2A). Aside from MEK1/MEK2, the MAPKKs MKK4 and MKK7 phosphorylate and activate the c-Jun N-terminal kinase (JNK) isoforms, MKK3 and MKK6 phosphorylate and activate the p38 isoforms, and MEK5 selectively activates ERK5. Depending on the cellular context, MKK4 may also contribute to the activation of the p38 pathway [6,7].

Structurally, MAPKKs are proteins of ~45-50 kDa that share 37-44% amino acid identity with MEK1/MEK2 in the kinase domain (Fig. 2B). MEK1 and MEK2 are themselves 86% identical in the catalytic domain. In addition to their kinase domain, MEK1 and MEK2 contain a strong leucine-rich nuclear export signal (NES) at their N-terminal extremity [8], a feature not found in other MAPKK family members. Contrary to MAP kinases, MAPKKs have very narrow substrate specificity. It is assumed, from lack of evidence to the contrary, that the MAP kinases ERK1/ERK2 are the only substrates of MEK1 and MEK2. However, the possibility that MEK1/MEK2 have other non-catalytic effectors cannot be excluded. For example, a recent study showed that MEK1 interacts with peroxisome proliferator-activated receptor γ (PPARγ) to induce its nuclear export and attenuate its transcriptional activity [9].

The high sequence identity between MEK1 and MEK2, and their significant similarity with MEK5 have important pharmacological implications. First, this explains why small molecule MEK1/2 inhibitors developed so far are non-selective with regard to MEK1 and MEK2 isoforms.

Although it is commonly believed that the two MAPKK isoforms are functionally equivalent, there is evidence, however, that they are regulated differentially and may not be interchangeable in all cellular contexts [10-13]. Intriguingly, it has been reported that activated MEK1 but not MEK2 induces epidermal hyperplasia in transgenic mice [14]. RNA interference and gene invalidation studies have also suggested that MEK1 and MEK2 may contribute differentially to tumorigenesis [15,16]. The physiopathological relevance of these observations to human cancer remains unclear. Second, it helps understand why the first-generation MEK1/2 inhibitors PD98059, U0126 and PD184352 were also found to inhibit MEK5 and the ERK5 MAP kinase pathway at higher concentrations [17,18]. Elucidation of the crystal structures of MEK1 and MEK2 has revealed that MEK5 share 83% amino acid identity with MEK1 in the PD184352-like inhibitor-binding pocket [19]. These MEK1/2 inhibitors have been used in thousands of papers and have proven extremely useful tools to investigate the biological functions of the ERK1/2 MAP kinase pathway. However, their inhibitory activity towards MEK5, albeit weaker, indicates that we should be cautious in the interpretation of data obtained at high concentrations of inhibitor.

## The ERK1/2 MAP kinase pathway is a key regulator of cell proliferation and survival

Multiple lines of evidence have implicated the ERK1/2 MAP kinase pathway in the control of cell proliferation [20]. First, ERK1 and ERK2 are activated in response to virtually all mitogenic factors. Second, several studies have reported that the mitogenic response to growth factors is correlated with their ability to induce sustained ERK1/2 activity [21-23]. Third, expression of kinase-dead mutants of ERK1 or anti-sense ERK1 RNA inhibited the activation of ERK1/ERK2 and exerted a dominant-negative effect on cell proliferation [24]. These early findings were confirmed by subsequent RNA interference-based studies showing that silencing of ERK1/ERK2 expression inhibits the proliferation of various cell types [25-27]. Fourth, treatment with small molecule inhibitors of MEK1/MEK2 was reported to inhibit the proliferation of a variety of cell types [28-30]. Reciprocally, expression of constitutively-active forms of MEK1 was sufficient to stimulate cell proliferation and relax growth factor dependency [31-33]. Further demonstration of the essential role of ERK1/2 signaling in cell proliferation was provided by gene invalidation studies in mice showing that loss of *Erk1 *or *Erk2 *gene function results in impaired proliferation of specific cell types [34-37].

ERK1/2 signaling is required for the progression of cells from the G0/G1 to S phase [20,38]. Activation of the ERK1/2 pathway is associated with induction of the positive cell cycle regulators cyclin D1 [39] and c-Myc [40], and with down-regulation of anti-proliferative proteins such as Tob1 [23], Foxo3a [41] and p21 [42]. In addition to its direct role in the cell division cycle, the ERK1/2 MAP kinase pathway also regulates cell growth by stimulating protein and nucleotide biosynthesis [20,43]. One mechanism by which the ERK1/2 pathway increases global protein translation is through phosphorylation and inactivation of tuberin (also known as TSC2), a negative regulator of the master growth regulator mammalian target of rapamycin (mTOR), resulting in increased mTOR signaling [44,45].

Studies in several experimental systems have highlighted the important role of the Raf-MEK-ERK1/2 MAP kinase pathway in the control of cell survival [46,47]. Early studies have shown that activation of the ERK1/2 pathway prevents apoptosis induced by growth factor withdrawal, loss of matrix attachment or cytoskeletal disruption in cultured cells [48-51]. These findings were reinforced by genetic studies showing that loss of ERK1/ERK2 or MEK1/MEK2 induces cell death in various mouse tissues [37,52,53]. ERK1/2 signaling promotes cell survival by repressing the expression or activity of pro-apoptotic Bcl-2 family proteins, such as Bim and Bad, and by inducing the expression of pro-survival members like Bcl-2 and Mcl-1 [47].

## Hyperactivation of the ERK1/2 MAP kinase pathway in cancer

**Figure 3:**
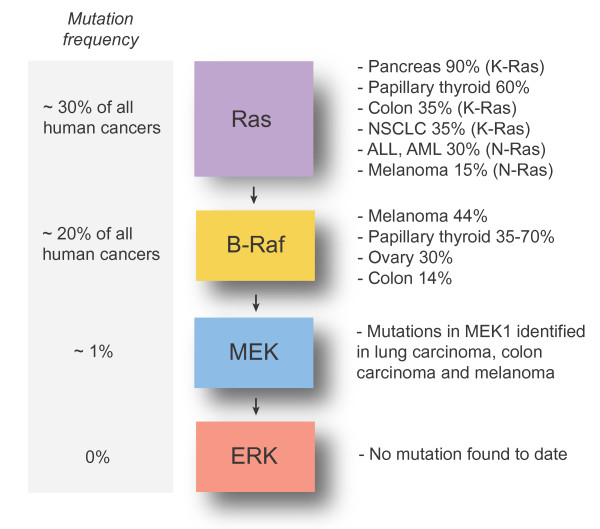
**Genetic alterations of the Ras-dependent ERK1/2 pathway in cancer**.

Given the central role of the Raf-MEK-ERK1/2 signaling pathway in cell proliferation and survival signaling, it is therefore not surprising that alterations in this pathway are highly prevalent in human cancer. Multiple genetic changes can lead to hyperactivation of the ERK1/2 pathway in cancer (Fig. 3). Aberrant activation of receptor tyrosine kinases such as the epidermal growth factor (EGF) receptor, as a result of gene amplification or gain-of-function mutations, is frequently observed in carcinomas and brain tumors [54,55]. Activating mutations in *RAS *genes, most often in *KRAS*, are found in ~30% of cancers and are generally acquired early in the tumorigenic process [56]. More recently, large-scale resequencing studies have revealed that *BRAF *is mutated in ~20% of all cancers and in more than 40% of melanomas [57]. The majority of these mutations are clustered in the kinase domain of B-Raf and lead to the stimulation of ERK1/2 activity in cells [58]. It is noteworthy that *RAS *and *BRAF *mutations are generally mutually exclusive in tumors, suggesting an epistatic relationship. Also, activating mutations in *MEK1 *gene are found at low prevalence in lung carcinomas, melanomas and colon carcinomas [59,60]. However, no mutation in the *ERK1 *or *ERK2 *gene has been reported to date in tumors. Consistent with these observations, numerous studies using clinical specimens have documented the hyperactivation of MEK1/MEK2 and ERK1/ERK2 in solid tumor and hematological malignancies [61,62].

Studies in cultured cells have revealed that expression of activated alleles of MEK1 or MEK2 is sufficient to deregulate the proliferation and trigger transformation of immortalized fibroblast and epithelial cell lines [15,31,32,63,64]. Orthotopic transplantation of mammary or intestinal epithelial cells expressing activated MEK1/MEK2 into mice induces the formation of aggressive tumors that progress up to the metastatic stage [15,64]. Similarly, expression of activated Raf mutants in various cell lines, including melanocytes, stimulates MEK1/2 and ERK1/2 signaling, and induces the formation of tumors in nude mice [65]. The oncogenic activity of the Raf-MEK-ERK1/2 pathway was further tested in transgenic mouse models. Transgenic expression of activated MEK1 in mouse skin induces hyperproliferative and inflammatory lesions and inhibits epidermal differentiation, mimicking features of squamous cell carcinomas [14,66,67]. In the same way, targeted expression of activated forms of C-Raf or B-Raf in various tissues of transgenic mice was shown to drive lung, skin, thyroid, and prostate tumorigenesis [65,68,69]. Importantly, deinduction of activated B-Raf expression in a conditional lung cancer mouse model leads to dramatic tumor regression concomitant to inactivation of ERK1/2 signaling, suggesting a dependency of B-Raf-induced lung tumors on the ERK1/2 pathway [70].

Pre-clinical pharmacological studies have demonstrated that blockade of the ERK1/2 pathway with small-molecule MEK1/2 inhibitors markedly restrains the proliferation of various carcinoma and leukemic cell lines by inducing cell cycle arrest and apoptosis [28,30,71,72]. In vivo studies further established that administration of orally available MEK1/2 inhibitors elicits significant tumor regression in mouse xenograft models [30,72-74]. The strategic position of MEK1 and MEK2 in the Ras-dependent ERK1/2 pathway in conjunction with a promising pre-clinical profile have provided strong rationale for the development of small-molecule inhibitors of MEK1/2 for chemotherapeutic intervention in cancer [62].

## Clinical development of MEK1/2 inhibitors

PD98059 was the first small-molecule inhibitor of MEK1/2 to be disclosed in 1995 [28]. Biochemical studies indicated that PD98059 inhibits the activity of both MEK1 and MEK2 isoforms, but fails to inhibit a panel of other Ser/Thr kinases [75,76]. Two other potent inhibitors of MEK1/2, U0126 [77] and Ro 09-2210 [78], were subsequently identified in cell-based assays. None of these compounds was moved to clinical evaluation because of their pharmaceutical limitations. However, PD98059 and U0126 have proven to be invaluable academic research tools to investigate the role of the ERK1/2 MAP kinase pathway in normal cell physiology and disease.

**Figure 4:**
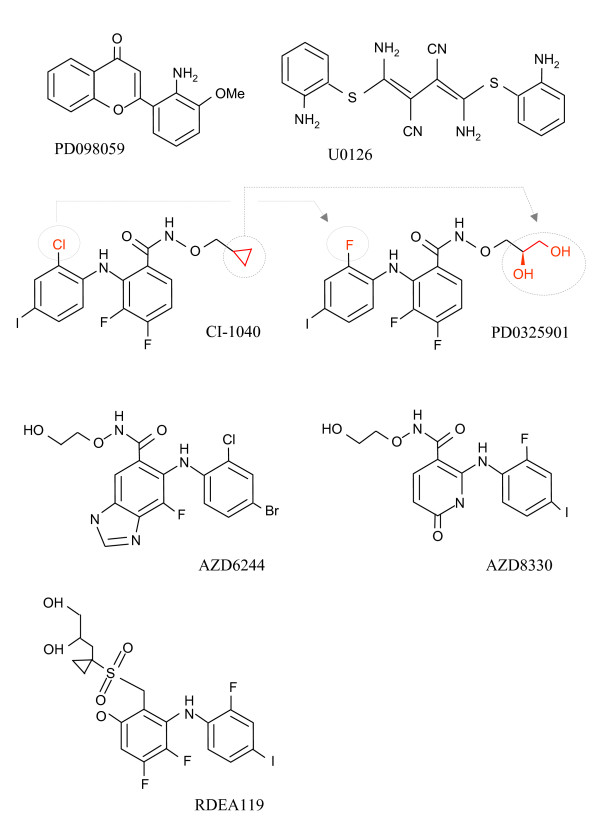
**Chemical structures of small molecule MEK1/2 inhibitors**.

To date, eleven MEK1/2 inhibitors have been tested clinically or are currently undergoing clinical trial evaluation (Table 1). The chemical structures of some of these inhibitors are given in Fig. 4.

**Table 1:** Small molecule MEK1/2 inhibitors in clinical trials

Inhibitor	Company	Phase	Status
CI-1040	Pfizer	Phase II	Development stopped
PD0325901	Pfizer	Phase I/II	Development stopped
AZD6244	Array BioPharma/ AstraZeneca	Phase II	In progress
GDC-0973	Exelixis/ Genentech	Phase I	In progress
RDEA119	Ardea Biosciences/ Bayer	Phase I/II	In progress
GSK1120212	GlaxoSmithKline	Phase I/II	In progress
AZD8330	Array BioPharma/ AstraZeneca	Phase I	In progress
RO5126766	Hoffmann La Roche	Phase I	In progress
RO4987655	Hoffmann La Roche	Phase I	In progress
TAK-733	Millenium Pharmaceuticals	Phase I	In progress
AS703026	EMD Serono	Phase I	In progress

### CI-1040 (PD184352)

The benzhydroxamate derivative CI-1040 (Pfizer) was the first MEK1/2 inhibitor to enter clinical trials [79]. CI-1040 is a potent (IC_50 _of 17 nM on purified MEK1) and highly selective inhibitor of MEK1 and MEK2 that was identified by screening a library compound with an in vitro ERK1 reactivation assay [30]. Similar to PD98059 and U0126, CI-1040 and its analogs inhibit MEK1/2 in a non-ATP and non-ERK1/2 competitive manner. Structural studies have revealed that CI-1040-related analogs bind into a hydrophobic pocket adjacent to but not overlapping with the Mg-ATP binding site of MEK1 and MEK2 [19]. Binding of the inhibitor induces a conformational change in unphosphorylated MEK1/2 that locks the kinase into a close catalytically inactive form. This binding pocket is located in a region with low sequence homology to other kinases (except for MEK5), which explains the high selectivity of these compounds and their noncompetitive kinetics of inhibition. In pre-clinical studies, CI-1040 was shown to inhibit the growth of colon carcinomas by as much as 80% in mouse xenograft models [30]. Importantly, antitumor activity was achieved at well-tolerated doses and correlated with a reduction in the levels of phosphorylated ERK1/2 in excised tumors.

A phase I study of orally administered CI-1040 was undertaken in 77 patients with advanced cancers [79]. Results of this study indicated that the compound was well tolerated at doses resulting in a median 73% inhibition of phospho-ERK1/2 expression in tumor biopsies. About 60% of patients experienced adverse effects, mostly grade 1 or 2, with no patient having drug-related grade 4 events. The most common toxicities included diarrhea, asthenia, rash, nausea, and vomiting. Interestingly, one patient with pancreatic cancer achieved a partial response with significant symptomatic improvement that lasted 12 months, and 19 additional patients suffering from a variety of cancers had disease stabilization lasting 4 to 17 months. This encouraging study provided the first demonstration that MEK1/2 can be inhibited in vivo in humans, and the first evidence of clinical activity for this class of agents. On this basis, a phase II study was initiated in 67 patients with advanced breast, pancreatic, colon and non-small cell lung cancers [80]. Unfortunately, results of this trial were disappointing. No patient achieved a complete or partial response, and stabilization of disease (median of 4.4 months) was observed in only 8 patients. The insufficient antitumor activity, poor solubility and low bioavailability of CI-1040 precluded further clinical development of this compound.

### PD0325901

The CI-1040 structural analogue PD0325901 (Pfizer) is a second-generation MEK1/2 inhibitor with significantly improved pharmaceutical properties [81]. Optimization of the diphenylamine core and modification of the hydroxamate side chain imparted PD0325901 with increases in potency, solubility and bioavailability. PD0325901 has an IC_50 _value of 1 nM against purified MEK1/MEK2, and inhibits the proliferation of various tumor cell lines at subnanomolar concentrations (100-fold more potent than CI-1040) [62,72]. In vivo studies have demonstrated that PD0325901 potently inhibits the growth of human tumor xenografts bearing activating mutations of B-Raf, concomitant with suppression of ERK1/2 phosphorylation [72]. The growth of Ras mutant tumors was also inhibited partially.

The clinical activity of PD0325901 was first evaluated in a phase I-II study of 35 patients with advanced solid tumors employing a dose-escalating design [82,83]. Doses ≥ 2 mg BID efficiently suppressed ERK1/2 phosphorylation (average of 84%) and Ki67 expression (average of 60%) in tumor biopsies. Anticancer activity of PD0325901 was evaluated from 27 assessable patients. Two partial responses were observed in melanoma patients, while 8 patients achieved stable disease lasting 3-7 months [84]. The phase I study was extended and clinical activity was documented by 3 partial responses in melanoma patients and 24 cases of disease stabilization (22 melanoma and 2 non-small cell lung cancer) in 66 patients [85]. However, PD0325901 was associated with more severe toxicity than CI-1040, including blurred vision as well as acute neurotoxicity in patients receiving more than 15 mg BID of the drug. The clinical development of this drug has been discontinued in 2008.

### AZD6244 (ARRY-142886)

The benzimidazole derivative AZD6244 (Array BioPharma/AstraZeneca) is another second-generation potent inhibitor of MEK1/MEK2 [86]. AZD6244 selectively inhibits purified active MEK1 and MEK2 with an IC_50 _of 14 nM by a mechanism not competitive with ATP. In cellular assays, the compound inhibits basal and growth factor-stimulated phosphorylation of ERK1/2 with IC_50 _concentrations < 40 nM, and exerts antiproliferative effects on tumor cell lines harboring *BRAF *or *RAS *mutations [86-88]. AZD6244 has demonstrated potent dose-dependent antitumor activity against a panel of mouse xenograft models of colorectal, pancreatic, liver, skin, and lung cancer [86-89]. Inhibition of tumor growth was found tocorrelate with the reduction of phospho-ERK1/2 levels in tumors. Based on promising pre-clinical activity, AZD6244 was advanced into clinical development.

A phase I clinical trial was undertaken to assess the safety, pharmacokinetics and pharmacodynamics of AZD6244 in 57 patients with advanced cancer [90]. Results of this study showed that the 50% maximal tolerated dose (100 mg BID) was well tolerated with skin rash being the most frequent and dose-limiting toxicity. Most other adverse events were of grade 1 or 2. Notably, 7 patients developed transient and reversible blurred vision, an adverse effect also observed with PD0325901. A strong reduction in ERK1/2 phosphorylation (mean inhibition of 79%) was observed in tumor biopsies. Nine patients showed disease stabilization lasting for at least 5 months.

Preliminary results from four randomized phase II clinical trials of AZD6244 have been recently reported. In a first study, AZD6244 was compared to the alkylating agent temozolomide in advanced melanoma patients. Antitumor activity of AZD6244 was observed, but there was no significant difference in progression-free survival between the two treatment arms [91]. A second study compared the efficacy of AZD6244 with the antimetabolite pemetrexed as second- or third-line treatment of patients with non-small cell lung cancer. Again, the study showed evidence of single agent antitumor activity, but failed to demonstrate a difference for the primary disease progression endpoint [92]. In a third study, AZD6244 was compared to capecitabine in patients with metastatic colorectal cancer who had failed prior irinotecan and/or oxaliplatin regimens. Similarly, no difference was observed between the two treatments in the number of patients with disease progression [93]. Finally, the results of a phase II study of AZD6244 in patients with advanced or metastatic hepatocellular carcinoma were recently reported. The study was stopped prematurely due to the lack of radiographic response [94]. Other phase II trials are currently ongoing in a variety of tumor types.

### GDC-0973 (XL518)

GDC-0973 (Exelixis/Genentech) is a potent, selective, orally active inhibitor of MEK1/2 with an IC_50 _of <1 nM in vitro [95]. In cellular studies, the compound inhibits ERK1/2 phosphorylation at subnanomolar concentrations, and exerts antiproliferative effects in multiple tumor cell lines harboring *KRAS *or *BRAF *mutations. In vivo pharmacodynamic studies have shown that a single oral dose of GDC-0973 inhibits phospho-ERK1/2 in tumors for up to 48 hours, translating into potent inhibition of tumor growth in human xenograft models. Notably, GDC-0973 appears to have reduced activity in the brain, which may reduce the potential of central nervous system side effects. A phase I dose-escalating study of GDC-0973 was initiated in subjects with solid tumors. Preliminary results from 13 patients indicates that GDC-0973 is well tolerated with no drug-related serious adverse events being reported [96]. One patient with non-small cell lung cancer had stabilization of disease for 7 months and continues on treatment. Another phase I trial of GDC-0973 in combination with the phosphatidylinositol 3-kinase (PI3K) inhibitor GDC-0941 is planned.

### RDEA119 (BAY 869766)

RDEA119 (Ardea Biosciences/Bayer) is another orally available, allosteric inhibitor of MEK1/2 [97]. In vitro, RDEA119 selectively inhibits MEK1 (IC_50 _of 19 nM) and MEK2 (IC_50 _of 47 nM) in a non-ATP competitive manner. Cellular assays showed that RDEA119 potently inhibits ERK1/2 phosphorylation (IC_50 _from 2.5 to 16 nM) and cell proliferation in a panel of human cancer cell lines. In vivo, RDEA119 exhibits potent antitumor activity in xenograft models of human melanoma, colon and epidermal carcinoma. Interestingly, pharmacodynamic studies have revealed that the compound has low central nervous system penetration. RDEA119 is currently being evaluated as single agent in a phase I study in advanced cancer patients, and in a phase I/II study in combination with the multikinase and Raf inhibitor sorafenib.

### GSK1120212

GSK1120212 (GlaxoSmithKline) is an orally available, selective inhibitor of MEK1/2 with reported antitumor activity in mouse xenograft models [98]. A phase I study of GSK1120212 was undertaken in 2008 in patients with solid tumors and lymphoma. Preliminary evaluation of 6 patients treated at four dose levels indicates that GSK1120212 is well tolerated with no dose-limiting toxicity reported so far [98]. Dose escalation is ongoing. Two other phase I/II studies of GSK1120212 have been recently initiated in subjects with relapsed or refractory leukemias, and in combination with everolimus in patients with solid tumors.

### OTHER MEK1/2 INHIBITORS

Five other MEK1/2 inhibitors are currently being evaluated in phase I clinical trials in advanced cancer patients. These are AZD8330 (Array BioPharma/AstraZeneca), RO5126766 and RO4987655 (Hoffmann La Roche), TAK-733 (Millenium Pharmaceuticals) and AS703026 (EMD Serono). Other novel MEK1/2 inhibitors such as RO4927350 and RO5068760 have recently been reported but have not yet passed the pre-clinical stage [99,100].

## Concluding remarks and challenges

Despite strong rationale for the clinical development of drugs targeting the ERK1/2 MAP kinase pathway in cancer, the effectiveness of this approach in cancer therapy remains to be validated. The first and only inhibitor of the ERK1/2 pathway that has received regulatory approval for the treatment of advanced renal cell carcinoma and hepatocellular carcinoma is the Raf inhibitor sorafenib (Nexavar) [101]. However, sorafenib is a multikinase inhibitor that also inhibits the vascular endothelial growth factor and platelet-derived growth factor receptor tyrosine kinases, as well as Flt-3 and c-Kit receptors. To what extent the inhibition of Raf signaling contributes to the clinical activity of the drug is not clear. Future clinical trials of more selective Raf inhibitors will help determine whether blocking the pathway at the level of Raf is a clinically viable approach. Inhibitors of MEK1/2 are highly selective for their targets. However, results from the first clinical trials have been disappointing. New MEK1/2 inhibitors with improved pharmaceutical properties and reduced central nervous system activity are promising and results of ongoing trials are anxiously awaited.

As for other targeted therapies, several outstanding questions remain to be addressed before MEK1/2 inhibitors join the arsenal of anticancer drugs. Which patients are more likely to benefit from MEK1/2 inhibitors? Pre-clinical studies suggest that patients harboring activating mutations in *RAS *or *BRAF *genes are better candidates for treatment with these kinase inhibitors. Thus, selection of appropriate patient populations based on genetic lesions or validated biochemical markers will be critical for future clinical trial evaluation. Is the therapeutic efficacy of MEK1/2 inhibitors hampered by dose-limiting toxicity? The ubiquitous involvement of the ERK1/2 MAP kinase pathway in cellular responses has raised concern about the potential toxicity of drugs blocking this pathway. The ocular toxicity observed with PD0325901 and AZD6244 suggests the existence of mechanism-based adverse effects. Interestingly, new MEK1/2 inhibitors such as GDC-0973 and RDEA119 have reduced activity in the brain, which may increase their therapeutic window. What are the most rationale and best combination therapies with MEK1/2 inhibitors? The multigenetic nature of advanced cancers suggests that MEK1/2 inhibitors will likely find their therapeutic utility in combination with other targeted agents or conventional cytotoxic drugs. Pre-clinical studies have shown that PI3K pathway activation, through *PIK3CA *activating mutations or *PTEN *loss of function, significantly decreases the response of *KRAS *mutant cancer cells to MEK1/2 inhibitors [102]. Importantly, simultaneous inhibition of the ERK1/2 and PI3K pathways was found to exert a marked synergistic effect on tumor regression [102,103]. These observations have provided a strong rationale for the combination of MEK1/2 and PI3K inhibitors in cancers that harbor concurrent activating mutations in these signaling pathways. In that context, Merck and AstraZeneca have recently announced their plan to collaborate in testing a combination therapy of AZD6244 and the Akt inhibitor MK-2206 in cancer [104]. Finally, is the acquisition of resistance mutations in MEK1/MEK2 going to limit the clinical utility of these small molecule inhibitors? A recent study has reported the identification of a resistant *MEK1 *mutation in a metastatic tumor that emerged in a melanoma patient treated with AZD6244 [105]. Therefore, it may prove necessary to target other components of the ERK1/2 pathway in patients who develop resistance or, eventually, to combine MEK1/2 inhibitors with Raf inhibitors to slow down the emergence of resistance. A phase I/II study of RDEA119 in combination with the multikinase Raf inhibitor sorafenib is currently ongoing.

## Competing interests

The authors declare that they have no competing interests.

## Authors' contributions

Both authors participated in drafting and editing the manuscript. Both authors read and approved the final manuscript.
